# Cholera

**Published:** 1849-08

**Authors:** 


*Cholera.* By N. Ward, M. D., Burlington, Vt.—This disease appeared several times in the northern province of Ceylon during the time I was resident there. About three years ago it prevailed extensively, and was of a severe character. In the treatment of it, some relied on calomel and opium in large doses. I derived the greatest benefit from a combination of calomel, opium, camphor, and sugar of lead. I tried acetate of morphia and prepared chalk, with peppermint. This relieved the pain, but did not stop the progress of the disease, except in mild cases. In some cases I gave sugar of lead and opium, and sometimes camphor, omitting the calomel. This treatment frequently arrested the discharges, but less surely than with calomel; and the disease was very apt to return in a short time. The tre ment where calomel was used was altogether the most satisfactory.

It did not seem to me important or desirable to produce salivation. For an adult, in a case of severity, I generally gave at one dose, calomel gr. x, p. opii gr. ij, camphor gr. v, acet. plumbi gr. ij, ground well together. This mixture was given in all stages of the complaint, from the incipient diarrhœa to the state of collapse, varying the dose according to the severity of the case. One dose was generally sufficient; if not, it was repeated. No food or drink was allowed till the patient was convalescent, and then with caution. Drinks did not relieve the thirst complained of by the patients, but seemed to keep up the discharges. There were instances in which the disease, after it had been apparently arrested, was brought back by the use of a cup of gruel. Care was taken not to give any thing that would decompose the acetate of lead. If the calomel did not act on the bowels in due time after the evacuations were stopped, and especially if pain in the abdomen came on, it was the practice to give a mild cathartic with some carminiative.

This plan of treatment I adopted ten years ago, and have had every reason to be satisfied with it. It secured the confidence of the community to a greater extent than any other. People were desirous of procuring doses of the medicine to a greater extent than we were able to supply them, to keep by them for use in the event of an attack. I heard of many persons whose lives were saved by this precaution. The cholera appeared to me to be as much under the control of medical treatment as any active disease we have to deal with.

The danger in this disease appears to be owing mostly to the rapid depletion that takes place in it. From some cause, to us hitherto unknown, the body is rapidly deprived of a large part of its fluids, poured out through the lining membrane of the alimentary canal and the skin; and though the character of the discharges is not well understood, nor that of the disordered condition by which they are produced, I have allowed myself to conjecture that the cholera may be more nearly allied to some forms of hemorrhage than to the ordinary bowel complaints. However this may be, have not the modes of treatment that have been reported as successful, been such as are adapted to produce action or change of action in the capillary system, and thus prevent the further reduction of the circulating mass?—*American Journal Medical Sciences.*

